# ROV assessment of mesophotic fish and associated habitats across the continental shelf of the Amathole region

**DOI:** 10.1038/s41598-021-97369-2

**Published:** 2021-09-13

**Authors:** Rio E. Button, Denham Parker, Vivienne Coetzee, Toufiek Samaai, Ryan M. Palmer, Kerry Sink, Sven E. Kerwath

**Affiliations:** 1 Department of Biological Sciences, University of Cape Town, Rondebosch, 7700 South Africa; 2 Department of Forestry, Fisheries and the Environment, Cape Town, 8000 South Africa; 3 Department of Biodiversity and Conservation, University of the Western Cape, Bellville, Cape Town, South Africa; 4 South African Institute for Aquatic Biodiversity, Somerset Street, Makhanda, 6139 South Africa; 5 South African National Biodiversity Institute, Rhodes Drive, Newlands, 7700 South Africa; 6 Institute for Coastal and Marine Research, Nelson Mandela University, Summerstrand, Gqeberha, 6001 South Africa

**Keywords:** Biodiversity, Community ecology, Conservation biology, Ichthyology, Ecology, Marine biology

## Abstract

Understanding how fish associate with habitats across marine landscapes is crucial to developing effective marine spatial planning (MSP) in an expanding and diversifying ocean economy. Globally, anthropogenic pressures impact the barely understood temperate mesophotic ecosystems and South Africa’s remote Amathole shelf is no exception. The Kei and East London region encompass three coastal marine protected areas (MPAs), two of which were recently extended to the shelf-edge. The strong Agulhas current (exceeding 3 m/s), which runs along the narrow shelf exacerbates sampling challenges. For the first time, a remotely operated vehicle (ROV) surveyed fish and their associated habitats across the shelf. Results indicated fish assemblages differed between the two principle sampling areas, and across the shelf. The number of distinct fish assemblages was higher inshore and on the shelf-edge, relative to the mid-shelf. However, the mid-shelf had the highest species richness. Unique visuals of rare *Rhinobatos ocellatus* (Speckled guitarfish) and shoaling *Polyprion americanus* (wreckfish) were collected. Visual evidence of rhodolith beds, deep-water lace corals and critically endangered endemic seabreams were ecologically important observations. The ROV enabled in situ sampling without damaging sensitive habitats or extracting fish. This study provided information that supported the Amathole MPA expansions, which extended protection from the coast to beyond the shelf-edge and will guide their management. The data gathered provides baseline information for future benthopelagic fish and habitat monitoring in these new MPAs.

## Introduction

Understanding how fish associate with habitats across marine landscapes is crucial to developing effective conservation and sustainability strategies^1^. If fish assemblages are associated with specific habitats and environmental features, then this information can be used for fish distribution projections^2^. This understanding is valuable for marine spatial planning (MSP), where information from different models can help strategize to mitigate the effects of anthropogenic pressures^3^. Anthropogenic pressures are rapidly altering marine ecosystems, including the scarcely understood mesophotic (between 30 and 150 m deep)^4^ and the ‘rariphotic’ (150–300 m depth) zone^5^. Examples of these pressures include fishing, ocean warming, acidification and pollution^6^. There is some evidence that the mesophotic zone provides refugia from these pressures in the tropics^7^ however, there is no comparable support in temperate zones^8^ where mesophotic biotic surveys are generally rare^9^.

Access to sampling the mesophotic zone is increasing as technological underwater video techniques advance^10^. Previously, visual biotic surveys were focused within regular scuba depths (typically up to 30 m) and more expensive techniques such as using submersibles focused on the deep sea^4^. With the development of Remotely Operated Vehicles (ROVs), submersibles and Autonomous Underwater Vehicles, non-destructive video sampling can be conducted across the mesophotic zone^11^. Some fish and mobile invertebrate species may actively avoid ROVs, which can compromise taxonomic resolution from underwater video surveys^12^. Despite these limitations, ROV videos provide a permanent record of fish abundance, composition and associated habitat^13^, allowing for a better understanding of the distribution of fish and habitat through the mesophotic zone^10,14^ without causing damage to the habitat or extracting species^15^. This makes it an ideal method for sampling areas of potential conservation value or protected areas^15^.

**Figure 1:**
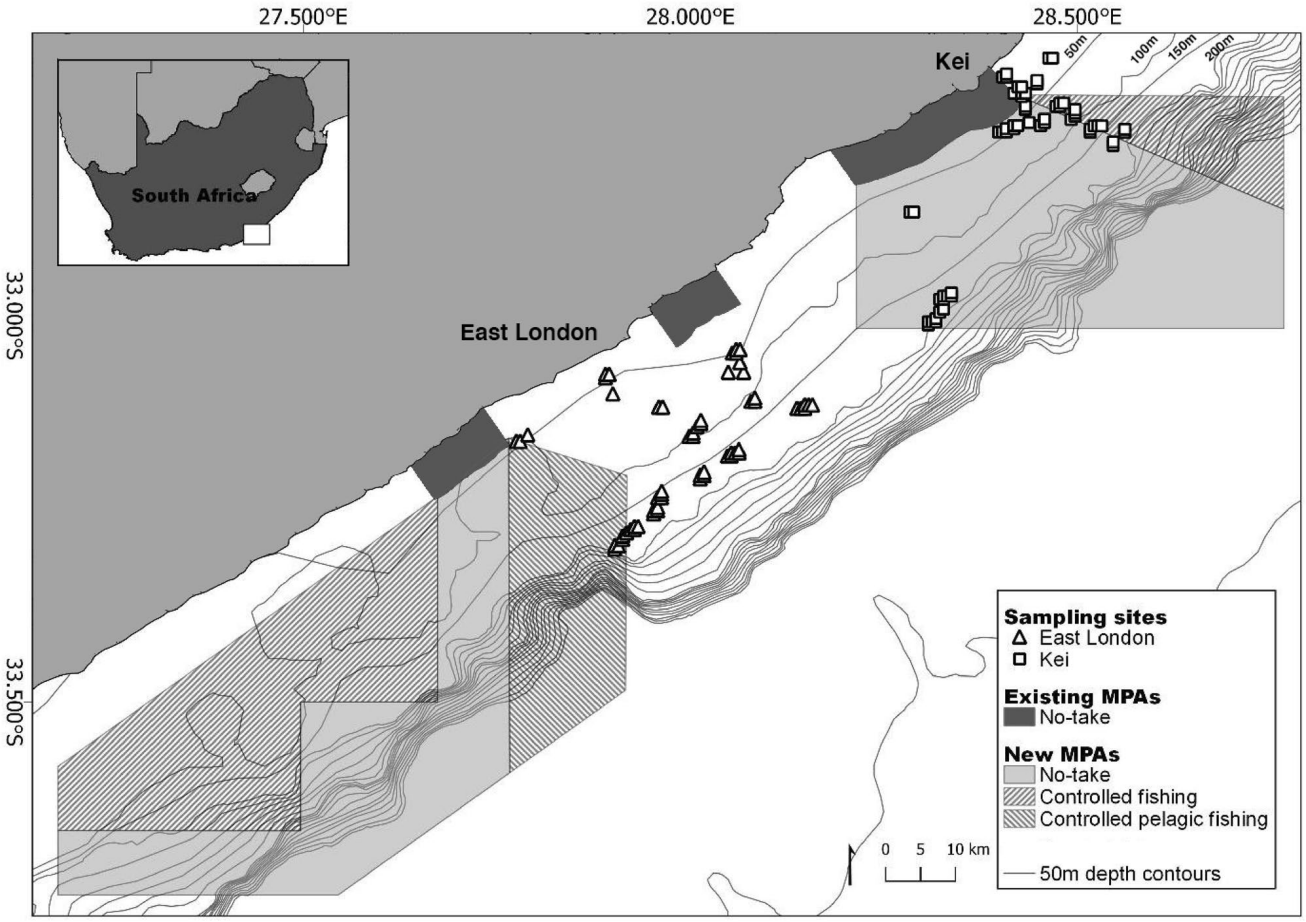
Study area, the Amathole continental shelf, on the south coast of South Africa where the East London (triangles) and Kei (squares) sampling sites were located. The 50 m bathymetric depth contours; existing MPAs, which were declared as official reserves in 1984 and later proclaimed as MPAs in 2011; and the new MPAs, proclaimed in 2019 are indicated. The MPAs are divided into zones with different restrictions namely (1) no-take; (2) controlled fishing, where extraction and harvesting of marine life is allowed with restrictions and limitations; and (3) controlled pelagic fishing, where only pelagic linefishing of specific species is permitted. Map created in QGIS ver. 3.8 (https://qgis.org/en/site/^56^) using shapefiles provided by the South African National Biodiversity Institute (https://www.sanbi.org/^64^) and sampling sites locations generated by this study.

There is a global commitment to increasing marine protected area (MPA) coverage^16^. In 2019, South Africa expanded MPA coverage from 0.4 to 5% of its Exclusive Economic Zone by increasing the number of MPAs from 21 to 41^17^. The new MPAs are focused on offshore protection^18^ and aim to be representative of offshore ecosystems and mitigate pressures from government planned ocean economy growth and industrialisation^19^. The new MPAs include the offshore expansion of existing MPAs. The Amathole region on the East coast of South Africa is considered an endemism hotspot^20^ and these areas were made reserves in 1984 and later MPAs in 2011^21^. The new Amathole Offshore MPA extends protection to the mesophotic ecosystems in the region for the first time^22^ (Fig. 1).

The Amathole region is a transition zone between two of the six defined marine Ecoregions of South Africa: the Agulhas (South Coast) and Natal (East Coast) Ecoregions^18^. The Agulhas Ecoregion is characterised by warm temperate waters, the widest margin of the country’s continental shelf (up to 240 km) and several reef complexes that hold the highest number of the country’s endemic fish species^15^. Distinguishing features of the Natal Ecoregion include subtropical waters, a narrow continental shelf (5–50 km), high riverine input, steep shelf-edges with numerous incising canyons, complex oceanographic patterns (upwelling cells and cyclonic eddies) driven by the dominant Agulhas current^15^. Apart from our surveys, which were prioritised to inform MPA placement prior to increasing coverage in 2019^17^, the area is largely unexplored as it has treacherous sea conditions. Strong currents persist throughout the area due to the Agulhas current being funnelled nearshore by the narrow continental shelf^23^. Furthermore, increased regulation on fisheries, particularly the commercial linefish sector, in response to depletion^24,25^ resulted in a substantial decrease in commercial fishing effort in the Amathole area over the past two decades^26,27^. Subsequently, commercial landings data have decreased to such an extent that it is no longer a reliable source of information on fish distribution and abundance in the area, and alternative data collection methods are required.

The aim was to visually explore and describe fish fauna and their associated benthic habitat as well as to determine which environmental variables (benthic biotic, substrate, and relief habitat variables as well as depth, distance from shore, and principal sampling areas) best explain patterns of fish distribution, abundance, and assemblage composition. To survey benthopelagic fish and their associated habitat, an ROV ran transects. We quantitatively surveyed fish species diversity and relative abundance in relation to depth, distance from shore and benthic habitat to provide a baseline assessment and assess the potential of ROVs as a sampling tool in a region characterised by strong currents. The study also represents the first visual survey of the capture site of the first coelacanth^28^. The outcomes of this study are aimed to support and guide marine spatial planning and conservation in the region.

## Results

A total of 54 h of footage was collected from 42 ROV transect dives (Supplementary Table S1) which translates to a total of 117 sampling sites (see “Methods” for how sampling sites were determined). From the 1829 MaxN observations made, a total of 65 fish species from 49 genera and 31 families were recorded (Supplementary Table S2), and 98% of observed fish were able to be identified to species level.

**Figure 2:**
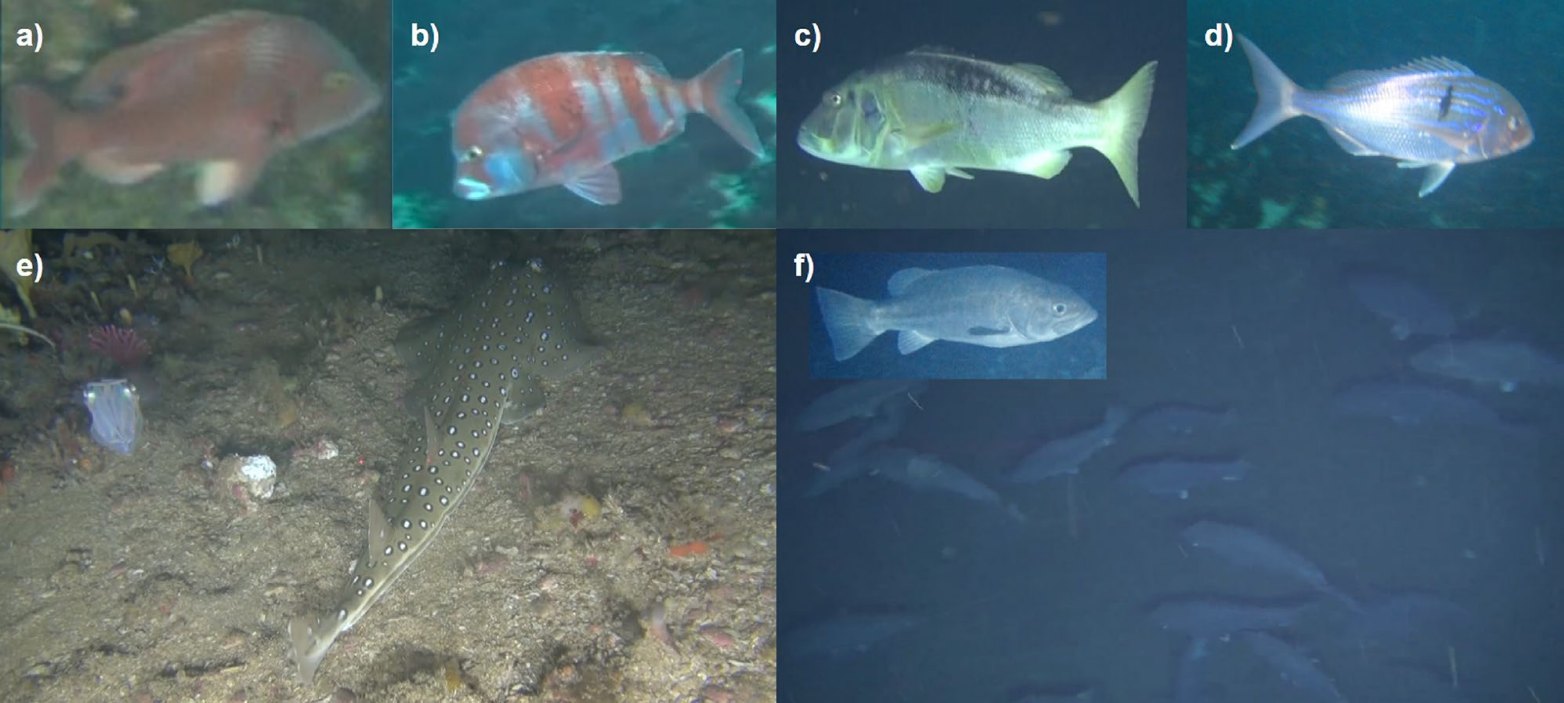
Fish images extracted from the ROV transect videos. Fish species that were endemic to South Africa include **(a)** *Chrysoblephus cristiceps* (dageraad) (critically endangered), **(b)** *Chrysoblephus gibbiceps* (red stumpnose) (endangered), and **(c)** *Petrus rupestris* (red steenbras) (endangered). While **(d)** *Polysteganus undulosus* (seventy-four) was critically endangered and endemic to southern Africa. A living **(e)** *Rhinobatos ocellatus* (speckled guitarfish) and shoaling **(f)** *Polyprion americanus* (wreckfish) were recorded on video for the first time.

**Figure 3:**
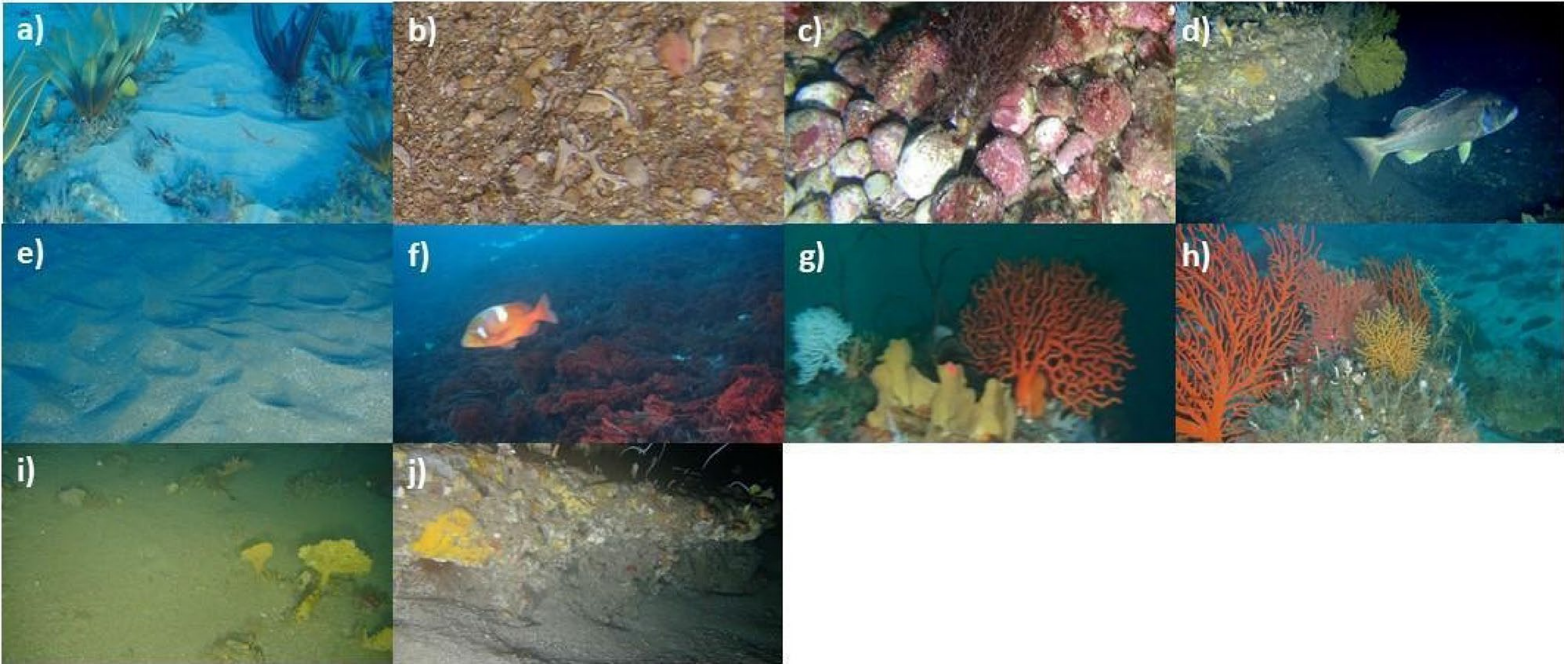
Images of dominant habitat for substrate clusters: **(a)** sand, **(b)** rubble, **(c)** rhodoliths and **(d)** rock. Biota clusters included: **(e)** no biota, **(f)** algae, **(g)** soft corals & sponges and **(h)** fan coral; and relief clusters **(i)** flat and **(j)** low, were extracted from the ROV transect videos.

Eight morphospecies groups were identified as taxonomic species names could not be determined for these groups. There was not enough visual detail in the footage to discern the specific species from their close relatives (e.g., members of Congridae). Interestingly, the morphospecies ‘lab suez’ was likely a species from the genus *Liopropoma*, which taxonomic experts hypothesised was a yet to be described species or species morph. In these cases, the species were given a unique identifier. The smallest fish species we were able to reliably identify was ~8 cm, was a *Nemanthias carberryi* (threadfin goldie). In all but one instance, it was possible to identify the families to which the morphospecies belonged. Of the species observed 32 were endemic to southern Africa and 14 to South Africa. Species diversity per sample site ranged from 0 to 19, average species richness (± standard deviation (s.d)) was 5.70 (± 4.28) and mean total species abundance (± s.d.) was 15.63 (± 16.28). The most frequently observed families were Sparidae (17 spp.), Serranidae (7 spp.), Labridae (4 spp.), and Triglidae (1 spp.) (Supplementary Table S2). The ROV allowed us to observe rare large, overexploited and critically endangered endemic seabreams including *Polysteganus undulosus* (Seventy-four) and *Chrysoblephus cristiceps* (Dageraad) as well as endangered *Chrysoblephus gibbiceps* (Red stumpnose) and *Petrus rupestris* (Red steenbras)^24,25^ (see Fig. 2a–d). Three endangered *Sphyrna lewini* (Scalloped hammerhead) were observed on a single transect that had a median depth of 90m. This study collected unprecedented observations of *Polyprion americanus* (Wreckfish) schooling as well as *Rhinobatos ocellatus* (Speckled guitarfish), which were both listed as data deficient by the IUCN red list^30^ (Fig. 2e,f). Rare habitat types, including rhodolith beds, sponges, and deep-water lace corals were also documented (Fig.3 and Supplementary Table S3).

The number of unique habitat variables observed included 12 substrata, 25 benthic biotas, and 4 reliefs (Supplementary Table S3). The most abundant substrate observed was rock (43%), the most abundant benthic biota was fan coral (soft coral) (23%), and ‘flat’ (48%) was the most abundant form of relief. Rhodolith substrate was documented between 33 and 64 m deep, with individual agglomerates being approximately 3–12 cm in diameter. Encrusting coralline algae were observed to a depth of 80 m. This was the deepest photosynthesising organism observed, which may be an indication of the maximum depth to which photosynthesis occurs in the region. Notable biotic habitats frequently observed included deepwater lace coral and sponge gardens. Habitat clustered into four substrate clusters, four biotic clusters and two relief clusters. Substrate clusters were dominated by sand, rhodoliths, rubble (coarse biogenic rubble), and rock respectively. No specific biota type dominated the benthic biota clusters, but lace corals, sponges, algae and sea fans were abundant at various depths while the relief clusters were dominated by flat and low relief (Fig. 3 and Supplementary Table S4).

**Figure 4:**
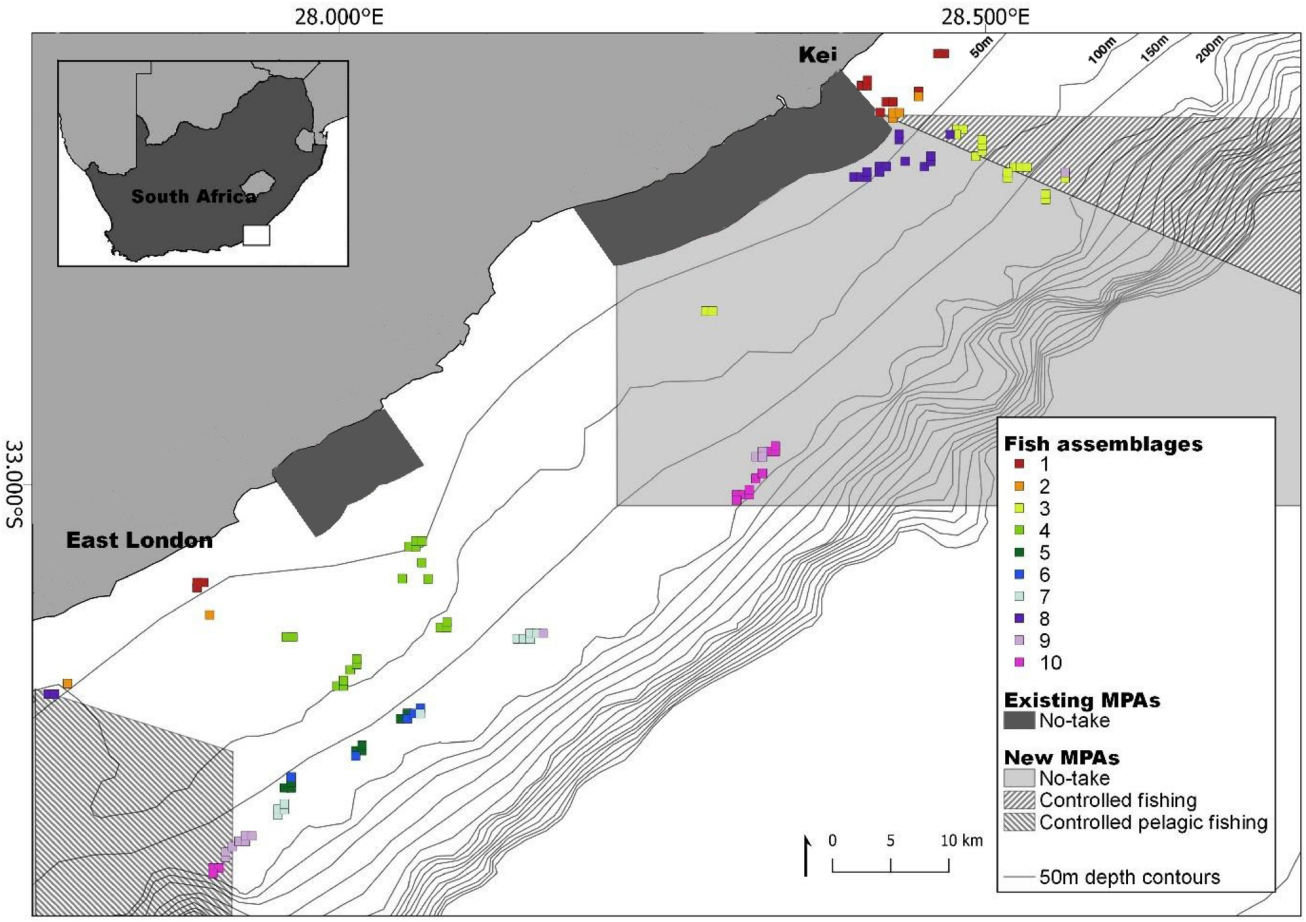
Spatial distributions of the fish assemblages on the Amathole continental shelf, located on the south coast of South Africa. Fish assemblages are clusters of species determined using a Multivariate Regression Tree that includes depth, substrate clusters, distance from shore and the two broad areas of East London and Kei. These fish assemblages are labelled 1 to 10. The 50 m bathymetric contours as well as existing and new Marine Protected Areas (MPAs) are indicated. The existing MPAs were declared as official reserves in 1984 and later proclaimed as MPAs in 2011 and the new MPAs were proclaimed in 2019. The MPAs are divided into zones with different restrictions namely (1) no-take, (2) controlled fishing, where extraction and harvesting of marine life are allowed with restrictions and limitations, and (3) controlled pelagic fishing, where only pelagic linefishing of specific species may occur. Map created in QGIS ver. 3.8^56^ using shapefiles provided by the South African National Biodiversity Institute^64^ and fish assemblages generated in R statistical software^59^(CRAN ver. 4.0.2).

**Figure 5:**
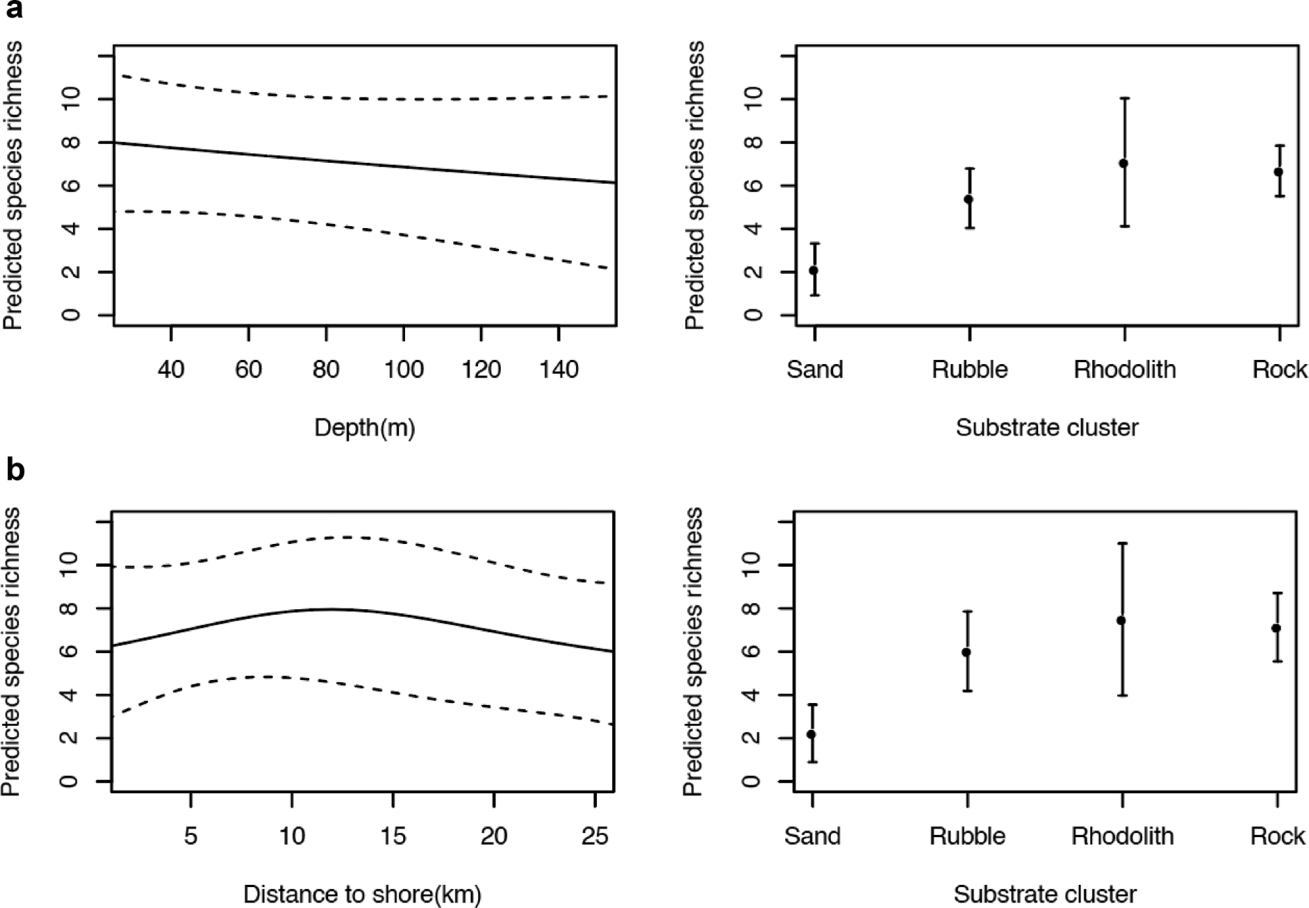
Generalized Additive Models (GAMs) for species richness in relation to **(a)** depth and **(b)** distance to shore and substrate cluster, which was the benthic explanatory variable with the lowest p-value. In the line graphs, the solid lines represent the predicted abundance or probability of presence per grid cell, and the dashed lines illustrate 95% confidence intervals. In the habitat category plots, dots represent the predicted probability of presence and whiskers illustrate 95% confidence intervals. Graphs created in R statistical software^59^ (CRAN ver. 4.0.2).

**Figure 6:**
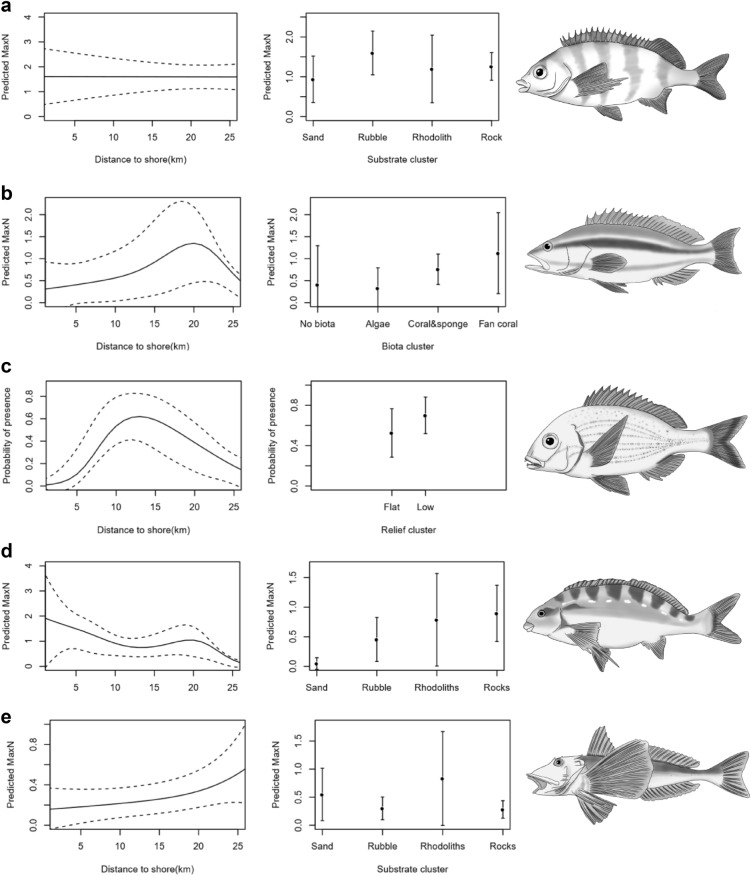
Generalized Additive Models (GAMs) for the five most abundant species **(a)** *Cheilodactylus pixi,* **(b)** *Serranus knysnaensis*, **(c)** *Pterogymnus laniarius,* **(d)** *Chirodactylus brachydactylus*, **(e)** *Chelidonichthys capensis* predicted abundance (for solitary species **a**,**b**,**e,d**) and presence (for shoaling species c) in relation to distance to shore and the benthic explanatory variable with the lowest p-value. In the line graphs, the solid lines represent the predicted abundance or probability of presence per grid cell, and the dashed lines illustrate 95% confidence intervals. In the habitat category plots, dots represent the predicted probability of presence and whiskers illustrate 95% confidence intervals. Graphs created in R statistical software^59^ (CRAN ver. 4.0.2). Illustrations were drawn and copyright permissions granted by Isabella Foulis.

Mean (± s.d) of species diversity was similar for the Kei and East London areas, 49 (± 0.43) and 47 (± 0.45), respectively. The important predictor variables for fish abundance data (in descending order) were substrate, distance from shore, depth, and principal sampling area (Kei and East London). These variables were included in the Multivariate Regression Tree (MRT) which resulted in the formation of ten unique fish assemblages. The fish assemblages initial split was explained by depth, the split occurred at 94 m (Supplementary Fig. S2). Species composition overlapped between assemblages (Supplementary Fig. S3). Assemblages were patchily distributed, however, similar assemblages tended to occur spatially close together—there were four fish assemblages unique to East London while one was unique to Kei (Fig. 4). Fish assemblages also varied along the shelf gradient, with higher fish assemblage diversity on the inshore and shelf-edge relative to the mid-shelf. Mean (± s.d.) species richness inshore (<100 m depth) was 58 (± 0.31) while offshore (>100 m depth) was 17 (± 0.44). When species richness was examined, distance from shore and depth were found to be co-linear, but the former was the better predictor of species richness. With distance from shore as a predictive variable, species richness peaked in the middle of the continental shelf (approx. 12 km from the shore). Species richness declined linearly with increasing depth. Substrate was the only significant variable for predicting species richness, and higher species richness was associated with consolidated substrates, rhodolith and rock (Fig. 5 and Supplementary Table S4). Substrate was the most important benthic habitat predictor for three of the five most abundant species: *Cheilodactylus pixi*, *Chirodactylus brachydactylus* and *Chelidonichthys capensis*. The formers abundance was associated with rubble while the abundance of the latter two was associated with rhodoliths. Of the remaining two species, *Serranus knysnaensis* abundance peaked in fan coral biota and *Pterogymnus laniarius* was most frequently observed in low relief areas (Fig. 6 and Supplementary Table S5).

## Discussion

With the use of ROVs, this exploratory study provided the first assessment of deeper mesophotic reefs, their benthopelagic ichthyofaunal assemblages and associated benthic habitats in the Amathole region of South Africa. This environment was previously inaccessible to scientific surveys. This study revealed an abundance of seabream (Family: Sparidae) species including *Polysteganus undulosus*, *Chrysoblephus gibbiceps*, *Pterogymnus laniarius*, and *Argyrozona argyrozona*. Their abundance is most encouraging as in 2000 South Africa’s traditional linefishery was declared in a state of emergency and the urgent need to rebuild the many overexploited linefish stocks was recognised^29,31^. After this species-specific management plans began being drawn up and were implemented in the mid-2000’s^29,31^. Study highlights included the first footage of *Rhinobatos ocellatus*, as well as the shoaling behaviour of *Polyprion americanus—*both species were classified as data deficient by the IUCN redlist^28^. *Rhinobatos ocellatus* was only known from three bycatch specimens^32^, despite their distinctive appearance^32,33^. Typically, *Polyprion americanus* was solitary and only known to aggregate off the coast of Brazil to spawn^34^. Their shoaling on the Amathole shelf-edge above deep-water corals suggests that the area might be a spawning habitat for this species; deep-water coral habitats are known to provide nurseries and spawning grounds for many species^35^. The benthic habitats sampled across the Amathole shelf were generally diverse and sometimes patchy in distribution. These findings contribute to the little that is known about South Africa’s offshore continental shelf biodiversity^36^.

The most important predictor of the five most abundant fish species differed, only *Chirodactylus brachydactylus* and *Chelidonichthys capensis* had their most important predictor in common, which was distance from shore. Hard substrate was an important predictor for *Cheilodactylus pixi*, *Chirodactylus brachydactylus*, and *Chelidonichthys capensis*, the latter two species were associated with rhodoliths while the former was associate with rubble. Substrate influences the seabed relief as well as the biota that can grow in a particular area, which could explain the relative importance of this variable. Biota was the most important predictor of *Serranus knysnaensis* abundance, which is a sedentary species^37^ and was commonly observed sheltering from strong currents behind fan corals. This observation was further justified with fan coral habitats having the highest MaxN for this species. Soft corals such as fan coral have been shown to provide shelter, a source of food or a surface on which epiphytic food was sourced^38^. Lastly, relief was the most important predictor for *Pterogymnus laniarius*. Low relief increased the probability of the species presence which aligns with the findings that *Pterogymnus laniarius* feed in sand and mud habitats as their stomach contents were mainly of soft sediment organisms^39^.

The fish assemblage structure was primarily split by depth at 94 m, this is deeper than the average depth (56 m) at which mesophotic coral ecosystem (MCE) fish communities have been reported to split into upper and lower mesophotic zones^40^. The primary assemblage split at 94 m likely reflects the transition between the photic and aphotic zone, which is presumed to be at ~80 m depth in this area (based on the observations of crustose coralline algae to this depth, beyond this depth photosynthesising organisms were not present).

Fish assemblages exhibited a level of spatial autocorrelation, with more overlap in assemblages occurring closer together while at a finer spatial scale, the number of fish assemblages differed across the continental shelf. The shelf-edge had the highest number of unique fish assemblages, followed by the inshore region and then the mid-shelf. Therefore, the number of fish assemblages were greatest where depth gradients were steeper. Depth is strongly correlated with other environmental gradients such as light, current strength and distance from shore^41,42^. Distance from shore was likely correlated with nutrient and turbidity parameters from river inputs^43,44^. We hypothesised areas with higher environmental gradients contained more niches, resulting in more fish assemblages in such areas. This could explain why more fish assemblages occurred in areas with larger depth gradients.

Three unique fish assemblages situated on the shelf-edge were characterised by rare and endangered fish species; the species which characterised each of these assemblages were endangered *Petrus rupestris*, endangered *Chrysoblephus gibbiceps*, and vulnerable *Polyprion americanus*. Identifying important areas for these species was the first step towards protecting their habitat. Fish assemblages unique to East London and Kei respectively justify the offshore expansion of both MPAs in the region (Fig. 1). In this case, multiple MPAs allow access to marine resources from the urban centre (East London) while still protecting fish assemblages unique to the north and south. The offshore expansions would also protect the unique assemblages that occur in shelf-edge habitats.

When assessing species richness, consolidated substrates (rhodolith and rock) were found to hold higher fish diversity than unconsolidated substrates (sand and rubble). Rhodoliths are unattached agglomerates of non-geniculate coralline algae that can form extensive beds^45^. Both rock and rhodoliths provided surfaces of attachment for sedimentary benthic organisms like coral and algae^46,47^. These benthic organisms could have attracted species that feed on them as well as the fish species that have taken refuge in the structural complexity they provided or physical disturbances they buffered.

Species richness increased at the mid-shelf when the distance to shore was included in the model, however, when modelled by depth there was a decrease in species richness with increasing depth. Distance from shore was likely a better predictor of species richness than depth. Distance from shore provided a consistent gradient, yet was still strongly correlated with abiotic variables such as current strength and sedimentation deposition rates, which could have influenced fish distributions^44^. Conversely, depth’s predictive power may have been reduced due to high relief features causing large variation in depth range sampled within each sample site. For example, the range of depths sampled could be extreme where gradients were steep, such as inshore or at the shelf-edge. Due to this, the median of depth per sample site was included in the model. Due to the variation in depth per sample site, distance from shore was a better proxy for environmental factors than median depth per sample site in this study. As nearshore sampling sites were shallower and offshore sites were deeper it was not possible to separate the influence of depth from distance to shore. Most studies found depth to be the primary predictor of species richness^48–52^. One of these studies did show that following depth, distance to shore was the next most significantly related variable to assemblage structure^49^.

The mid-shelf peak in species richness, when using distance from shore, supported the mid-domain effect (MDE) hypothesis^53^. The MDE states that without environmental gradients, if species ranges were random (within a bounded geographical area) overlap between ranges would increase towards the middle of the area^53,54^. Thus, the species richness peak can be explained by its location in the middle of the geographic range of the continental shelf which is bounded by the shelf-edge and the shore. However, the shelf is not without environmental gradients so it does not meet the assumptions of the MDE^54^. This mid-shelf peak in species richness could be attributed to the overlap between photic and aphotic fish assemblages in this area. The photic zone extended to at least 80 m, indicated by the presence of photosynthesising organisms at this depth.

Despite the effectiveness of the sampling method for capturing habitats and enabling satisfactory taxonomic resolution of species with 98% of individual fish identified to species level, it became evident that some species we sampled shied away from the ROV. Other studies found that some species were deterred by ROVs (e.g., on South Africa’s Agulhas Bank^55^). A limitation of the method is that we may have missed small and cryptic species. The strong Agulhas current, sometimes exceeding 3 m/s, often hindered the ROVs ability to stop and examine points of interest. As extraction of data from the ROV footage was time-consuming, future visual surveys of this type will benefit from applying machine learning algorithms to reduce data extraction time so that underwater videos are more frequently and repeatedly utilised.

This study provided valuable information for MSP in the Amathole region through the identification of important habitats associated with fish distribution, abundance, and diversity data. Evidence that fish assemblages varied latitudinally (north-west to south-east), as well as from near shore to shelf-edge, supported the expansion of two MPAs in the Amathole region, providing latitudinal and shore-to-shelf-edge protection. The protection of the shelf-edge is especially important as it is critical habitat for endangered species such as *Petrus rupestris* and *Polyprion americanus*. Furthermore, this information provides a baseline for monitoring and management of the newly implemented MPAs in terms of their benthic biodiversity and fishery management objectives.

## Methods

### ROV transects

A Seaeye Falcon ROV (SAAB, system 12177) equipped with a Sub Sea Imaging 1Cam HD camera and three 3250 Lumen LED floodlights ran 42 transects in the mesophotic zone off the Kei River and East London between January and May 2017 (details of which can be seen in Supplementary Table S1). Transect locations were selected using two sources of information: (1) single and multibeam sonar data; and (2) local knowledge of recreational fishing locations. Using this information, transect locations were stratified according to depth (range: 30–170 m), seabed profile and, where multibeam sonar data were available, substrate type. Preference was given to locations that were in close proximity to known recreational fishing spots. Some dives were terminated early due to strong currents and technical difficulties.

In order to effectively operate the ROV in the strong Agulhas current, a 300 kg clump-weight system was incorporated. The ROV umbilical was connected along the clump-weight cable with 50 m of free tether between the weight and ROV. The clump-weight hung directly below the boat, which was equipped with Hamilton Jet engines that enabled live-boating in the strong current conditions.

When the current was strong, we aimed to carry out straight transects parallel to the shore, with the direction of the current (generally in a south-westerly direction), by positioning the boat to maintain a slow drift (<2 m/s) with the current. When the current was weak we would carry out several transects at the selected location in different directions. The ROV was flown below the boat at approximately 1 m above the seafloor in the direction of the current to maintain proximity with the clump-weight and avoid being “dragged”. This study was exploratory in nature, therefore, if a fish we could not immediately identify was seen we did change course in an attempt to get an identification, after which we resumed in the transect direction. A horizontal camera angle was generally maintained, with the exception of period 45° still photographs taken to analyse the benthic environment. The boat GPS continually logged the transect location as the ROV did not have an internal GPS system, and therefore the location accuracy of the transect is assumed to be less than 50 m away given the length of the tether between the weight and ROV.

### Analysis of video metrics and fish and habitat identification

The ROV transects GPS tracks were overlaid with 0.25 × 0.25 nautical mile grid (which is equivalent to a 463 × 463 m grid) in QGIS 3.8 (https://qgis.org/en/site/^56^). The footage that fell within each grid cell was determined, and cells that contained more than three minutes of bottom time footage were analysed as sample sites (Supplementary Fig. S1)—cells with less footage were discarded to avoid spurious results from under-sampling. On average there were two sample sites in each transect (Supplementary Table S1). Transects traversed patchy mosaics of diverse habitats, thus dividing transects into grid cells was a means of creating standardised units to define habitats and quantify fish species within. Transects were on average (± s.d.) 3.86 km (± 2.82) long, 3.51 km (± 3.29) apart (Supplementary Table S1), and 59 min (± 12 min) in duration. On average (± s.d.) sample sites were explored for 17 min (± 12 min). In total there were 117 sampling sites whose median depth ranged from 30 m to 162 m, with a mean (± s.d.) of 84 m (± 29) (Supplementary Table S1). Given the uncertainty of the ROV position relative to the boat position (introduced by the 50 m tether to the clump weight) 0.25 × 0.25 nautical mile was the smallest feasible resolution for analysis. Classifying habitat and quantifying fish per sample site allowed us to draw meaningful relationships between fish and habitat. Due to the patchy nature of habitats and larger areas used by more mobile fishes it was less accurate to draw associations between fish and the habitat observed directly below them and more meaningful patterns could be drawn from analysing footage per grid cell.

To quantify the benthopelagic ichthyofauna, the maximum number of individuals per species per frame (MaxN) was recorded within each sample site. MaxN is a conservative estimate of abundance, eliminating the possibility of recounting an individual fish within a sample site^57^. Fish were identified to the lowest possible taxa and every effort was made to identify large, conspicuous fish, in addition to small and cryptic species. Within each sample site, the depth and habitat (benthic biota cover, substrate and relief) were determined at five evenly spaced points in time. ROV videos and deployment data are archived in the African Coelacanth Ecosystem Programme database at the South African Institute for Aquatic Biodiversity and can be requested.

### Defining habitat

To classify habitat in terms of substrate, biota and relief we used the CATAMI classification system, a standardised vocabulary for identifying habitat from underwater imagery^58^. Rarely sampled habitats (n < 5 sample sites) were removed.

All statistical analyses were performed in R statistical software^59^ (CRAN ver. 4.0.2). To create representative levels of coarse structural habitat from the diversity of benthic habitats sampled, hierarchical clusters were created for each habitat type: substrate, biota and relief by means of the ‘NbClust’ library^60^. The final number of clusters for each habitat type was based on the following considerations. First the habitat composition of each cluster represented habitat types that were observed in the footage. Second each cluster was sufficiently sampled, i.e. n > 5 sample sites made up each cluster.

Habitat sampled was categorised into coarse structural habitat variables. This meant that potentially influential microhabitat detail was lost. However, if habitat variables were clustered on a finer scale it could have hindered efforts to extract habitat associations with fish species. Fish are motile and the boundaries between the environments they inhabit are not only vague but potentially dynamic. Fine-scale clusters would also reduce the number of sampling sites per cluster, which could affect the models’ ability to detect meaningful patterns.

### Fish assemblage structure

We investigated the spatial distribution of fish assemblages. A random forest was performed (by utilising the ‘randomForest’ library^61^) to robustly and accurately determine which habitat clusters and environmental variables were important for predicting fish species composition. The four most influential variables were substrate clusters, distance from shore, depth, and principal sampling area. These variables were included in a MRT, which split the grid cells similar in their species composition based on variable value thresholds. The percentage contribution of each species (with a greater than 5% frequency of occurrence) to the fish composition of each terminal group was calculated by means of the ‘mvpart’ library^62^. The distribution of the resulting ten fish assemblages was mapped with QGIS 3.8 (https://qgis.org/en/site/^56^).

Generalised additive models (GAMs) were used to investigate the influence that environmental variables had on benthopelagic ichthyofauna species richness, as well as species-specific models for the five most abundant species (we used these species because they had the most data and could thus produce more accurate results than other species). Collinearity was tested for between depth and distance to shore^63^. As they were found to be collinear, species richness was predicted in relation to habitat types and the variable depth or distance to shore independently. The habitat type with the lowest p-value was retained. We used the five most common species for species-specific models as they had the most data and would thus produce the most accurate results. These models utilized relative abundance (MaxN) with the exception of *Pterogymnus laniarius,* which was modelled using presence-absence to overcome zero-inflation as a result of schooling behaviour*.* Furthermore, distance to shore was retained as it explained more variation in the data than depth, and the habitat type with the lowest p-value was also retained.

## Supplementary Information

Supplementary Information.
