# Supplementary material for: ROV assessment of mesophotic fish and associated habitats across the continental shelf of the Amathole region
Source: Sci Rep. 2021 Sep 13;11:18171. doi: 10.1038/s41598-021-97369-2 (PMC8437978; doi:10.1038/s41598-021-97369-2)
Supplement: Supplementary file 1 — Supplementary Information. [file 41598_2021_97369_MOESM1_ESM.pdf]

ROV assessment of mesophotic fish and associated habitats across the continental shelf of the Amathole region.

Rio E. Button[1,*], Denham Parker[2,1], Vivienne Coetzee[1], Toufiek Samaai[1,2,3], Ryan M. Palmer[4], Kerry Sink[5,6], and Sven E. Kerwath[2,1]

[1] Department of Biological Sciences, University of Cape Town, Rondebosch, 7700, South Africa
[2] Department of Forestry, Fisheries and the Environment, Cape Town, 8000, South Africa.
[3] Department of Biodiversity and Conservation, University of the Western Cape, Bellville, Cape Town, South Africa
[4] South African Institute for Aquatic Biodiversity, Somerset Street, Makhanda, 6139
[5] South African National Biodiversity Institute, Rhodes Drive, Newlands, 7700, South Africa
[6] Institute for Coastal and Marine Research, Nelson Mandela University, Summerstrand, Port Elizabeth, 6001, South Africa.
[*] riobutton@gmail.com

Supplementary Table S1: Transect number with the associated number of sample sites, average sample site duration, dive distance, average depth, co-ordinates, and sampling date.

Transect number	Number of samples sites per transect	Average sample site duration (hh:mm:ss)	Dive distance (km)	Median depth (m)	Latitude	Longitude	Date
4	3	0:27:10	4.142	106.17	-33.28145463	27.91800723	1/24/2017
5	3	0:21:03	3.174	148.80	-33.29827912	27.90551366	1/24/2017
6	4	0:14:51	2.416	87.50	-33.23027414	27.96279802	1/24/2017
8	1	0:59:46	1.52	87.10	-33.05964407	28.06525623	1/26/2017
9	1	0:03:43	0.115	82.90	-33.07159265	28.04913791	1/26/2017
10	6	0:10:48	2.871	91.54	-33.11764877	28.14787229	1/26/2017
12	4	0:20:44	2.611	99.93	-33.27359014	27.92669482	1/29/2017
13	3	0:10:56	7.503	48.00	-32.71440891	28.42988511	1/30/2017
14	4	0:13:35	2.416	77.50	-32.73905338	28.49727507	1/30/2017
15	1	0:30:15	7.807	50.00	-32.75046372	28.4381568	1/30/2017
16	2	0:18:50	0.137	58.00	-32.69789672	28.44791433	1/30/2017
17	6	0:10:25	2.649	91.81	-33.17762808	28.05677282	1/31/2017
25	1	0:34:42	3.485	65.50	-33.15500303	27.78857538	2/5/2017
28	1	0:41:57	5.891	76.50	-33.10114263	27.89990609	2/5/2017
29	1	0:38:59	8.336	79.70	-33.13982192	28.01281661	2/7/2017
30	2	0:15:47	1.147	80.75	-33.13924156	28.01278736	2/7/2017
32	1	0:08:46	2.04	87.90	-33.07630654	28.063333	2/7/2017
33	4	0:16:26	2.212	84.51	-33.04663788	28.05702788	2/7/2017
41	4	0:14:02	1.773	88.68	-33.25012173	27.95743662	2/9/2017
42	4	0:12:33	1.763	88.56	-33.20525634	28.01680396	2/9/2017
43	3	0:23:21	1.648	78.87	-33.15416554	28.00209029	2/9/2017
49	3	0:21:20	7.672	46.67	-32.75858989	28.41765709	5/5/2017
50	1	0:15:59	7.004	36.00	-32.71044108	28.41657163	5/5/2017
52	3	0:18:26	1.693	87.33	-32.75639143	28.51894216	5/5/2017
59	2	0:27:30	1.015	80.90	-33.11841547	27.96328962	5/9/2017
71	3	0:10:56	9.92	54.73	-33.07624561	27.89279228	5/14/2017
74	2	0:28:45	1.185	49.30	-33.16247645	27.77724909	5/14/2017
77	3	0:19:55	2.052	85.40	-33.10901583	28.08228398	5/15/2017
84	4	0:15:40	1.706	61.50	-32.72626318	28.48049512	5/18/2017
85	3	0:32:17	1.755	59.33	-32.75004046	28.45619959	5/18/2017
86	4	0:13:50	3.066	57.75	-32.76182055	28.40363173	5/18/2017
87	2	0:16:40	1.068	46.50	-32.72926516	28.43212307	5/18/2017
91	2	0:16:24	7.608	88.50	-32.75440827	28.5311359	5/20/2017
92	2	0:35:09	4.469	94.50	-32.76716193	28.55294254	5/20/2017
93	2	0:15:24	8.544	86.50	-32.77772454	28.54589266	5/20/2017
94	2	0:15:24	7.193	53.50	-32.66525667	28.46452332	5/20/2017
95	3	0:10:33	5.992	32.00	-32.69078463	28.40653992	5/20/2017
96	2	0:17:55	7.934	39.00	-32.70424392	28.42488555	5/20/2017
97	2	0:15:25	9.051	88.00	-32.86596235	28.286861	5/20/2017
98	5	0:11:51	3.353	153.20	-33.00683021	28.3147916	5/21/2017
99	2	0:15:10	1.813	157.00	-32.99238106	28.32762237	5/21/2017
100	6	0:10:26	2.656	122.50	-32.97505223	28.33280062	5/21/2017

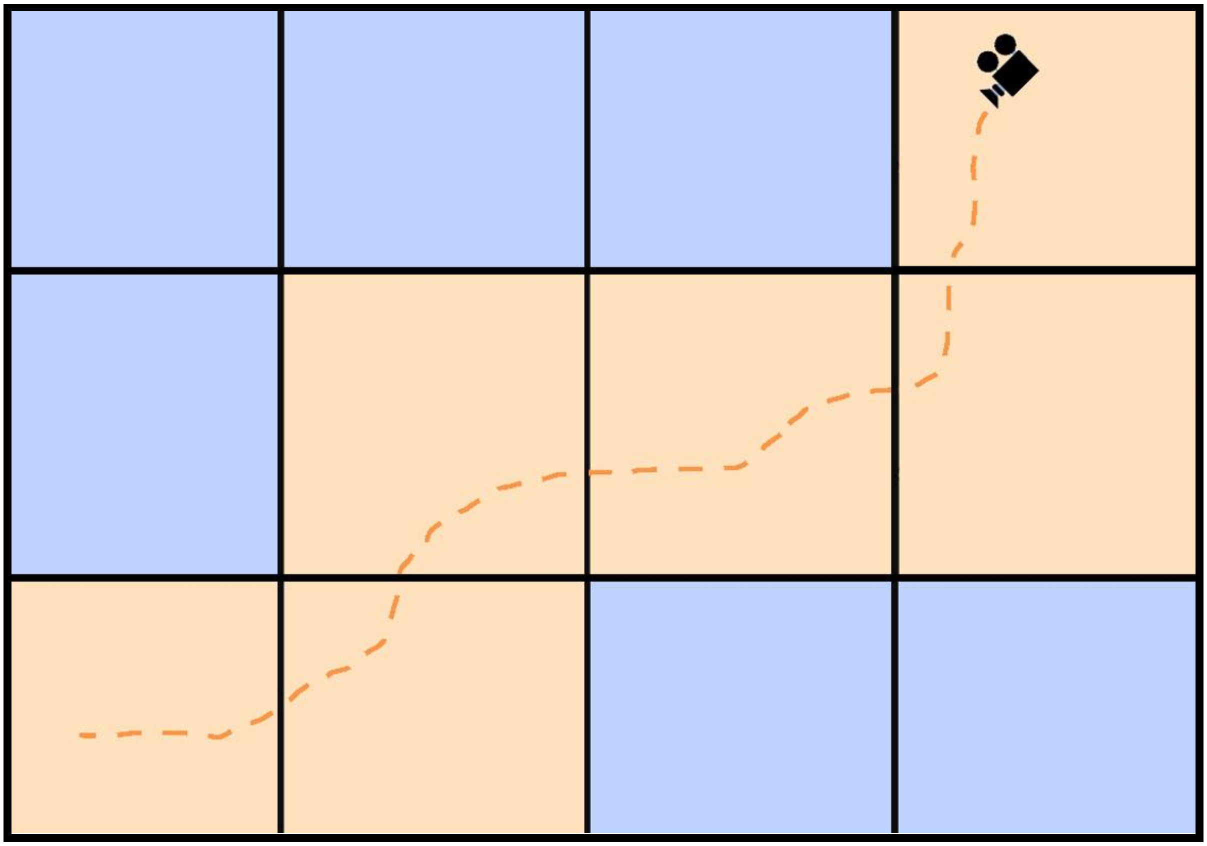

Supplementary Figure S1: An illustration of how a transect (orange dashed line) was divided up into grid cells (light orange squares), grid cells are 0.25 x 0.25 nautical miles (463 x 463 m grid). The footage that fell within each grid cell was determined, and cells that contained more than three minutes of bottom time footage were analysed as sample sites - cells with less footage were discarded so as to avoid spurious results from undersampling.

Supplementary Table S2: Fish species identified on the Amathole continental shelf during ROV transects. The percentage of sampling sites each species occurred in indicated the entire study region (all), the principal sampling areas Kei and the East London respectively. The maximum MaxN (maximum number of fish observed in a single video frame) for each species indicated Kei and East London sampling areas, respectively. Species endemic to southern Africa are indicated with an asterisk, while those endemic to South Africa are indicated by two asterisks.

Order	Family	Genus	Species	Common name	IUCN status	% of sample sites			Maximum Max N	
						All	Kei	East London	Kei	East London
Lamniformes	Odontaspididae	*Carcharias*	*taurus*	Spotted ragged tooth shark	VU	1%	2%	0%	1	0
Carcharhiniformes	Scyliorhinidae	*Haploblepharus*	*edwardsii*	Puffadder shyshark**	NT	1%	2%	0%	1	0
Carcharhiniformes	Scyliorhinidae	*Halaelurus*	*lineatus*	Lined catshark*	DD	1%	2%	0%	1	0
Carcharhiniformes	Sphyrnidae	*Sphyrna*	*lewini*	Scalloped hammerhead	EN	2%	0%	3%	0	2
-	-	-	-	Shark	NE	1%	0%	2%	0	1
Squatiniformes	Squatinidae	*Squatina*	*africana*	African angelshark	NT	3%	3%	2%	1	1
Rhinopristiformes	Rhinobatidae	*Rhinobatos*	*ocellatus*	Speckled guitarfish**	DD	1%	2%	0%	1	0
Rhinopristiformes	Rhinobatidae	*Rhinobatos*	*annulatus*	Lesser guitarfish*	LC	1%	0%	2%	0	1
Anguilliformes	Congridae	-	-	Conger	NE	1%	0%	2%	0	1
Gonorynchiformes	Gonorynchidae	*Gonorynchus*	*gonorynchus*	Beaked sandfish	LC	1%	0%	2%	0	1
Siluriformes	Ariidae	*Galeichthys*	*feliceps*	White sea catfish*	NE	1%	0%	2%	0	1
Aulopiformes	Synodontidae	-	-	Lizardfish	NE	2%	0%	3%	0	1
Aulopiformes	Synodontidae	*Congiopodidae*	*torvus*	Smooth horsefish*	NE	4%	3%	5%	1	1
Beryciformes	Berycidae	*Centroberyx*	*spinosus*	Short alfonsino**	NE	3%	3%	2%	3	5
Syngnathiformes	Centriscidae	*Macroramphosus*	*scolopax*	Slender snipefish	NE	4%	5%	2%	1	1
Scorpaeniformes	Scorpaenidae	-	-	Scorpionfish	NE	1%	2%	0%	1	0
Scorpaeniformes	Scorpaenidae	*Helicolenus*	*dactylopterus*	Bigscale scorpionfish	NE	2%	0%	3%	0	1
Scorpaeniformes	Scorpenidae	-	-	Jacopever	LC	12%	10%	14%	1	3
Scorpaeniformes	Triglidae	*Chelidonichthys*	*capensis*	Cape gurnard*	NE	35%	29%	41%	2	3
Labriformes	Labridae	*Bodianus*	*leucosticticus*	Lined hogfish	LC	5%	2%	8%	1	1
Labriformes	Labridae	*Coris*	*caudimacula*	Spottail coris	NE	22%	45%	0%	3	0
Labriformes	Labridae	-	-	Undifferentiable wrasse species’	NE	3%	2%	3%	1	1
Perciformes	Caesionidae	*Caesio*	*xanthonota*	Yellowback fusilier	LC	1%	0%	2%	0	1
Perciformes	Carangidae	*Cheilodactylus*	*fasciatus*	Redfingers*	NE	6%	10%	2%	1	1
Perciformes	Carangidae	*Seriola*	*lalandi*	Giant yellowtail	LC	2%	3%	0%	7	0
Perciformes	Chaetodontidae	*Chaetodon*	*marleyi*	Doublesash butterflyfish*	LC	1%	0%	2%	0	2
Perciformes	Cheilodactylidae	*Chirodactylus*	*brachydactylus*	Twotone fingerfin*	NE	36%	38%	34%	4	4
Perciformes	Cheilodactylidae	*Chirodactylus*	*grandis*	Bank Steenbras*	NE	9%	5%	12%	1	2
Perciformes	Cheilodactylidae	*Cheilodactylus*	*pixi*	Barred fingerfin*	NE	73%	67%	78%	5	10
Perciformes	Epinephelinae	*Epinephelus*	*marginatus*	Yellowbelly rockcod	VU	2%	3%	0%	1	0
Perciformes	Haemulidaea	*Pomadasys*	*striatus*	Striped grunter*	NE	1%	2%	0%	6	0
Perciformes	Oplegnathidae	*Oplegnathus*	*conwayi*	Cape knifejaw**	NE	13%	12%	14%	1	2
Perciformes	Parascorpididae	*Parascorpis*	*typus*	Jutjaw*	NE	4%	5%	3%	1	2
Perciformes	Pinguipedidae	*Parapercis*	*maritzi*	Sand diver**	NE	3%	7%	0%	1	0
Perciformes	Polyprionidae	*Polyprion*	*americanus*	Wreckfish	DD	3%	3%	2%	19	1
Perciformes	Sciaenidae	*Umbrina*	*canariensis*	Baartman	LC	1%	2%	0%	9	0
Perciformes	Serranidae	-	-	Rockcod	NE	1%	0%	2%	0	1

Perciformes	Serranidae	*Nemanthias*	*carberryi*	Threadfin goldie	LC	8%	16%	0%	20	0
Perciformes	Serranidae	*Epinephelus*	*chabaudi*	Moustache rockcod	LC	3%	2%	3%	1	1
Perciformes	Serranidae	*Pseudanthias*	*gibbosus*	One stripe goldie	LC	23%	24%	22%	8	28
Perciformes	Serranidae	*Serranus*	*knysnaensis*	Comber	NE	44%	26%	63%	2	2
Perciformes	Serranidae	*Meganthias*	*natalensis*	Gorgeous swallowtail	LC	2%	3%	0%	1	0
Perciformes	Serranidae	*Pseudanthias*	*squamipinnis*	Goldie	LC	3%	2%	3%	1	6
Perciformes	Sparidae	*Pachymetopon*	*aeneum*	Blue hottentot**	NE	32%	29%	34%	25	54
Perciformes	Sparidae	*Chrysoblephus*	*anglicus*	Englishman*	NT	4%	7%	2%	2	1
Perciformes	Sparidae	*Argyrozona*	*argyrozona*	Carpenter**	NT	9%	16%	3%	9	26
Perciformes	Sparidae	*Diplodus*	*cervinus*	Zebra	LC	1%	2%	0%	1	0
Perciformes	Sparidae	*Chrysoblephus*	*cristiceps*	Dageraad**	CR	8%	7%	8%	1	3
Perciformes	Sparidae	*Gymnocrotaphus*	*curvidens*	John brown**	LC	3%	5%	0%	1	0
Perciformes	Sparidae	*Porcostoma*	*dentata*	Dane*	LC	3%	5%	2%	1	1
Perciformes	Sparidae	*Spondyliosoma*	*emarginatum*	Steentjie**	NE	8%	16%	0%	4	0
Perciformes	Sparidae	*Chrysoblephus*	*gibbiceps*	Red stumpnose**	EN	18%	14%	22%	2	7
Perciformes	Sparidae	*Boopsoidea*	*inornata*	Fransmadam**	NE	15%	29%	0%	6	0
Perciformes	Sparidae	*Pterogymnus*	*laniarius*	Panga*	NE	41%	34%	47%	7	31
Perciformes	Sparidae	*Chrysoblephus*	*laticeps*	Roman*	NT	4%	9%	0%	5	0
Perciformes	Sparidae	*Chrysoblephus*	*lophus*	False englishman*	LC	3%	5%	2%	2	2
Perciformes	Sparidae	*Pagellus*	*natalensis*	Pandora	LC	2%	0%	3%	0	1
Perciformes	Sparidae	*Cheimerius*	*nufar*	Santer	NE	3%	3%	3%	1	1
Perciformes	Sparidae	*Petrus*	*rupestris*	Red steenbras**	EN	12%	7%	17%	1	3
Perciformes	Sparidae	*Polysteganus*	*undulosus*	Seventy-four*	CR	26%	28%	24%	11	23
Pleuronectiformes	Soleidae	*Cynoglossus*	*capensis*	Sand tonguefish*	NE	1%	0%	2%	0	1
Tetraodontiformes	Balistidae	*Sufflamen*	*bursa*	Boomerang triggerfish	NE	2%	2%	2%	1	1
Tetraodontiformes	Monacanthidae	-	-	Filefish	NE	1%	0%	2%	0	1
Tetraodontiformes	Monacanthidae	*Cantherhines*	*pardalis*	Honeycomb filefish	LC	1%	0%	2%	0	1

Supplementary Table S3: Biota, substrate, and relief habitats variables observed.

Substrate	Biota	Relief
Mud	Encrusting red calcareous algae	Flat
Fine sand	Laminate red algae	Low
Coarse sand	Erect fine branching brown algae	Moderate
Coral rubble	Erect fine branching red algae	High
Shell hash	Encrusting sponge	
Gravel	Erect form stalked sponge	
Pebble	Cup form sponges	
Cobbles	Erect form branching sponge	
Rhodoliths	Erect form laminate sponge	
Boulders	Erect form palmate sponge	
Rock	Erect form simple sponge	
	Massive form sponge	
	Branching fleshy arborescent coral	
	Non-fleshy arborescent coral	
	Fan coral	
	Whip corals	
	Bottle brush simple coral	
	Hydrocorals branching	
	Hydroids	
	Featherstars unstalked crinoids	
	Polychaete tubeworms	
	Brittle stars	
	Seastars	
	Zoanthids	
	No visible biota	

Supplementary Table S4: Composition of habitat clusters. Habitats with less than 5% frequency of occurrence were excluded.

Substrate clusters	Sand	Rubble	Rhodoliths	Rock
Shell hash	0%	11%	0%	0%
Rock	5%	21%	25%	83%
Rhodoliths	0%	0%	67%	0%
Mud	0%	9%	0%	0%
Coral rubble	8%	53%	0%	0%
Coarse sand	87%	6%	8%	17%
**Biota clusters**	**No biota**	**Algae**	**Coral & sponge**	**Fan coral**
Whip corals	0%	0%	23%	0%
No biota	100%	0%	13%	0%
Massive sponge	0%	0%	8%	0%
Fan coral	0%	0%	17%	100%
Erect sponge	0%	0%	8%	0%
Laminate sponge	0%	0%	23%	0%
Palmate sponge	0%	0%	8%	0%
Branching algae	0%	91%	0%	0%
Encrusting algae	0%	9%	0%	0%
**Relief clusters**	**Flat**	**Low**		
Flat	82%	26%		
Low	18%	60%		
Moderate	0%	14%		

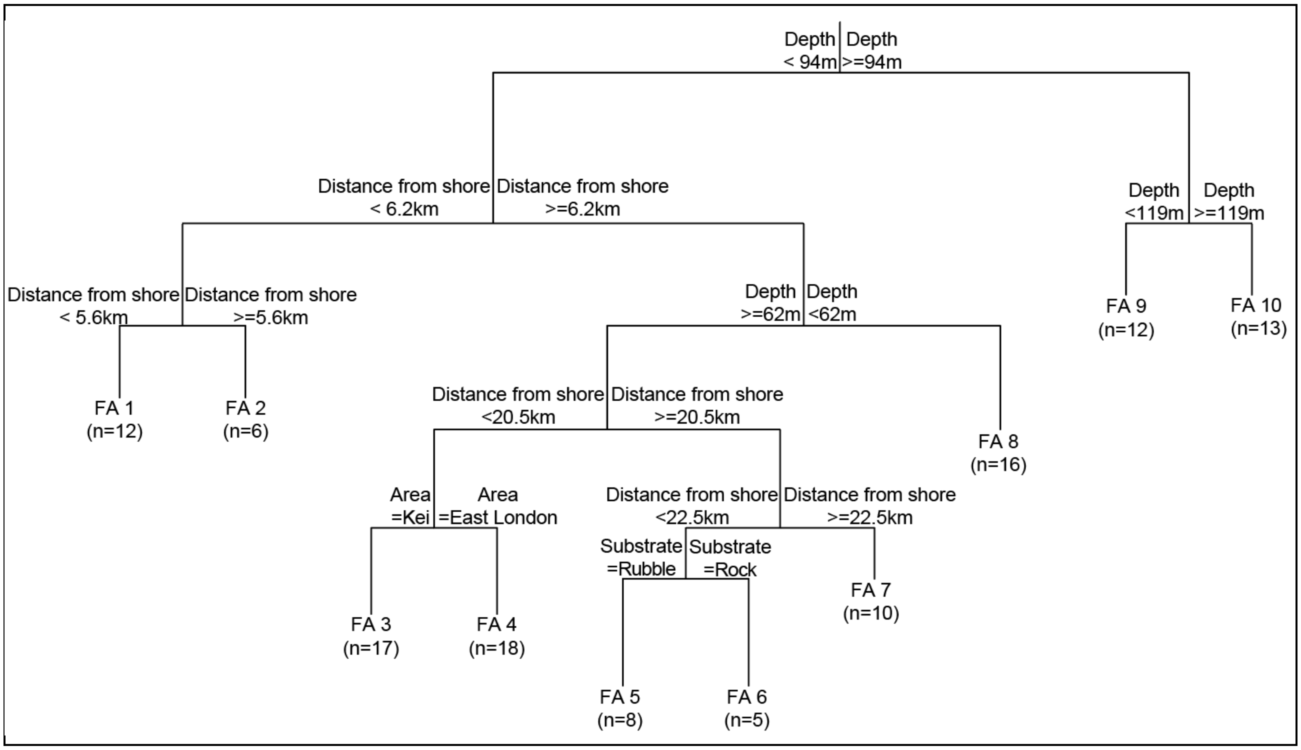

Supplementary Figure S2: Multivariate regression tree used to create the ten fish assemblages (FA) and their environmental explanatory variables. The number of grids cells each FA contains (n) is indicated. Diagram created in R statistical software[59](CRAN ver. 4.0.2).

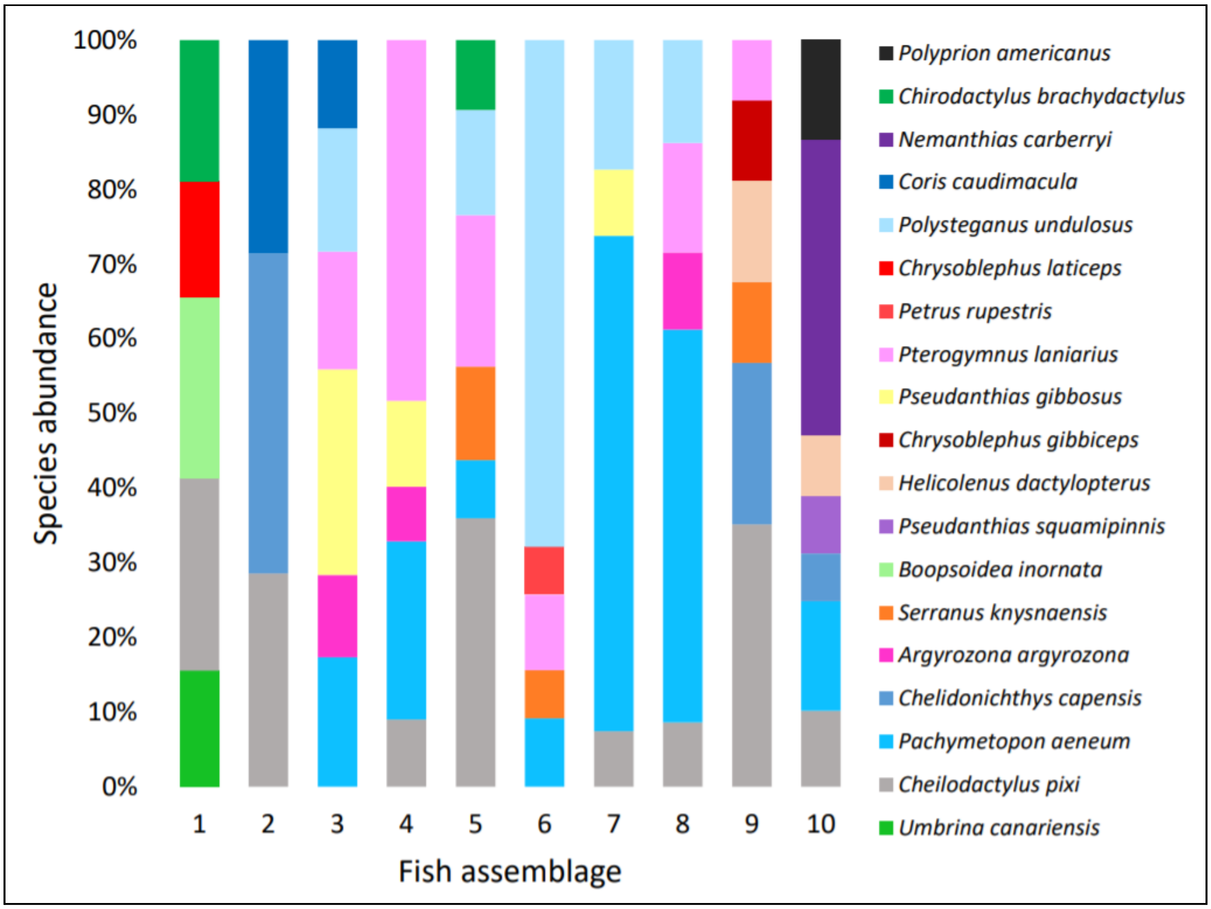

Supplementary Figure S3: Species composition of fish assemblage 1 to 10. Species with less than 5% occurrence per assemblage were excluded.

Supplementary Table S5: Generalized additive models diagnostics of species richness and and the five most common species predicted probability of abundance or presence in relation to depth and or distance from shore and their habitat category explanatory variable with the lowest p value (* indicate significant p values at the 0.05 level). Abundance was used for species richness and solitary species while presence was used for shoaling species.

**Response variables**	**Distribution**	**Explanatory variables**	**P value**	**Deviance explained**	**Degrees of freedom**
**Species richness**	Quasi-Poisson	Depth<br>Substrate	0,43<br>0,002*	15,50%	3
	Quasi-Poisson	Distance from shore<br>Substrate	0,32<br>0,002*	17,70%	3
Cheilodactylus pixi	Quasi-Poisson	Distance from shore<br>Substrate	982<br>553	2,81%	3
Serranus knysnaensis	Poisson	Distance from shore<br>Biota	491<br>367	22,50%	3
Pterogymnus laniarius	Binomial	Depth<br>Relief	0,001*<br>97	16,20%	1
Chirodactylus brachydactylus	Poisson	Distance from shore<br>Substrate	0,011*<br>0,012*	32,90%	3
Chelidonichthys capensis	Poisson	Distance from shore<br>Substrate	78<br>259	7,45%	3
